# Components of *Panax ginseng* and *Rhodiola rosea* regulate mitophagy via the SIRT1/3-PGC-1α-NRF2 pathway to improve myocardial ischemia-reperfusion injury

**DOI:** 10.3389/fphar.2026.1716078

**Published:** 2026-02-06

**Authors:** Ming Yao, Xiaoli Wang, Hongyu Wei, Lisha Wang, Dongze Zhang, Zheng Liang, Rong He, Yuan He, Lihong Jiang, Yingzi Cui

**Affiliations:** 1 Department of Cardiovascular Medicine, Affiliated Hospital of Changchun University of Chinese Medicine, Changchun, China; 2 College of Traditional Chinese Medicine, Changchun University of Chinese Medicine, Changchun, China; 3 College of Rehabilitation Medicine, Changchun University of Chinese Medicine, Changchun, China

**Keywords:** ginsenoside Rg1, salidroside, myocardial ischemia-reperfusion injury, mitochondrial function, mitophagy

## Abstract

**Introduction:**

The combination of ginsenoside Rg1 and salidroside (PRC) exhibits cardioprotective potential against myocardial ischemia-reperfusion injury (MIRI), yet its underlying mechanism remains unclear.

**Materials and Methods:**

An *in vivo* rat model of MIRI and an *in vitro* H/R model using H9c2 cardiomyocyte were established. PRC was administered, and its effects on myocardial injury, oxidative stress, mitochondrial function, and endothelial markers were evaluated. Key proteins in the SIRT1/3-PGC-1α-NRF2 pathway and mitophagy (Beclin 1, p62, PINK1, Parkin, TOM20) were analyzed by Western blot. The functional necessity of SIRT1/3 was validated using siRNA knockdown.

**Results:**

PRC reduced infarct size, ameliorated mitochondrial ultrastructure, and attenuated oxidative stress *in vivo*. *In vitro*, PRC enhanced cell viability, restored ATP and mitochondrial membrane potential, and suppressed ROS production ROS. Mechanistically, PRC activated the SIRT1/3–PGC-1α–NRF2 axis, normalized PINK1/Parkin expression, preserved mitochondrial content (indicated by restored TOM20 levels), and inhibited excessive autophagy (evidenced by downregulated Beclin1 and upregulated p62). Notably, silencing SIRT1 or SIRT3 abolished these protective effects, confirming their essential upstream regulatory roles.

**Conclusion:**

PRC attenuates MIRI by activating the SIRT1/3–PGC-1α–NRF2 pathway to modulate PINK1/Parkin-dependent mitophagy, thereby restoring mitochondrial homeostasis. Our study elucidates a novel mechanism underlying this natural product combination and highlights the SIRT1/3 axis as a promising therapeutic target for cardioprotection.

## 1 Introduction

Acute myocardial infarction (AMI) remains a leading cause of death worldwide. While timely reperfusion is essential to salvage ischemic myocardium, it paradoxically induces myocardial ischemia-reperfusion injury (MIRI), which significantly compromises its clinical efficacy (Neri et al., 2017). The pathogenesis of MIRI involves a complex cascade including calcium overload, oxidative stress, and profound mitochondrial dysfunction (Bai et al., 2021). Mitochondria are central to this process, as ischemia impairs oxidative phosphorylation, leading to ATP depletion, while reperfusion triggers a massive burst of reactive oxygen species (ROS), resulting in oxidative damage, opening of the mitochondrial permeability transition pore (mPTP), and ultimately, cardiomyocyte apoptosis (Sun et al., 2021; Schirone et al., 2022; Zhang et al., 2024). Furthermore, the critical role of PINK1/Parkin-mediated mitophagy, a selective form of autophagy for damaged mitochondria, has been increasingly recognized as a double-edged sword in MIRI, with its precise regulation being essential for cardioprotection (Vargas et al., 2023; Zhao X. et al., 2024). Therefore, developing strategies to protect mitochondrial function represents a promising therapeutic avenue for MIRI.

The multi-target properties of natural products make them suitable for addressing the complex pathogenesis of conditions like MIRI. *Panax ginseng C. A. Meyer* and *Rhodiola rosea L.* are two renowned herbs in traditional Chinese medicine with documented cardioprotective properties. These two herbs are, in fact, the principal components of the Shenhong Tongluo Formula, a traditional herbal formulation that has demonstrated clinical efficacy in alleviating post-PCI symptoms and limiting experimental myocardial infarction (Chang et al., 2013; Zhao et al., 2023). Ginsenoside Rg1 (Rg1), a primary active component of *P. ginseng*, and salidroside (Sal), the characteristic constituent of *Rhodiola rosea*, have individually exhibited efficacy in ameliorating MIRI through their antioxidant, anti-apoptotic, and anti-inflammatory activities (Wang et al., 2022; Chen et al., 2023; Zhou et al., 2024; Dong et al., 2025; Wang et al., 2025). Notably, emerging evidence suggests that both compounds can modulate mitochondrial homeostasis, with some studies implicating SIRT1-PGC-1α signaling in their protective mechanisms (Wang et al., 2022; Zhang et al., 2023).

Despite the preliminary validation of synergistic effects for the combination ginsenoside Rg1 and salidroside (PRC), several questions remain unresolved (Lai, 2022; Zhao, 2022). The precise molecular mechanisms by which this combination exerts its coordinated effects, particularly concerning mitochondrial quality control, are not fully understood. Furthermore, although SIRT1 and PGC-1α have been implicated in this process, the precise hierarchical relationship within the SIRT1/3-PGC-1α-NRF2 pathway has not been clearly established, particularly regarding the respective contributions and necessity of both SIRT1 and SIRT3. More fundamentally, it remains unknown whether this pathway functionally converges with the crucial quality control process of mitophagy to coordinate cardioprotection.

Thus, this study aimed to evaluate the protective effects of PRC against MIRI, with particular focus on its role in regulating mitochondrial quality control through the SIRT1/3-PGC-1α-NRF2 pathway. Using a combination of *in vivo* and *in vitro* models combined with genetic approaches, we sought to determine whether this pathway functionally interacts with PINK1/Parkin-mediated mitophagy. Furthermore, the essential upstream regulatory roles of both SIRT1 and SIRT3 were systematically validated using siRNA knockdown experiments.

## 2 Materials and methods

### 2.1 Main materials and reagents

Male Sprague-Dawley (SD) rats (weighing 300 ± 20 g, Certificate No. SCXK (Liao) 2020-0001) were obtained from Liaoning Changsheng Biotechnology Co. Ltd. (Liaoning, China).

Ginsenoside Rg1 (purity ≥98%, cat. no. AB1275) and Salidroside (purity ≥98%, cat. no. AB1838) were purchased from Alfa Biotechnology (Chengdu, China). 3-methylpurine (3-MA, purity ≥98%, cat. no. S24823) was obtained from Yuanye Bio-Technology (Shanghai, China). Dimethyl sulfoxide (DMSO, cat. no. D8418) was acquired from Sigma (Missouri, USA). 2,3,5-Triphenyltetrazolium chloride solution (TTC, cat. No. G1017), electron microscope fixative (cat. no. G1102), and protein-free rapid blocking solution (cat. no. G2052) were purchased from Servicebio Technology (Wuhan, China). Hematoxylin-eosin solution (HE, cat. no. B1000) was sourced from Baiqiandu Biotechnology (Wuhan, China). Rat thromboxane A2 (TXA2, cat. no. MM-0633R1), prostacyclin 2 (PGI2, cat. no. MM-0708R1), soluble thrombomodulin (sTM, cat. no. 20954R1), and soluble endothelial protein C receptor (sEPCR, cat. no. MM-0166R1) ELISA kits were obtained from Jiangsu Enzyme Exemption Industry (Jiangsu, China). CCK-8 detection kit (cat. no. IV08-100) was obtained from Invigentech (California, USA). ATP assay kit (cat. no. S0026) and ROS assay kit (cat. no. S0033S) were obtained from Beyotime Biotechnology (Shanghai, China). Mitochondrial membrane potential assay kit (cat. no. M8650) was purchased from Solarbio Science & Technology (Beijing, China). Lactate dehydrogenase (LDH, cat. no. A020-2-2), malondialdehyde (MDA, cat. no. A003-1-2), and superoxide dismutase (SOD, cat. no. A001-1-1) assay kit were sourced from Nanjing Jiancheng Bioengineering Institute (Nanjing, China). Prestained protein marker (cat. no. PL00001) was obtained from Proteintech (Wuhan, China).

### 2.2 Ethics and experimental animals

All animal protocols used in this study were approved by the Experimental Animals Ethics Committee of Changchun University of Chinese Medicine (Approval No. 2022491), which follows the guidelines established by the National Institutes of Health. In line with the ARRIVE guidelines, we implemented specific measures to minimize suffering. All surgical procedures were conducted under isoflurane anesthesia to ensure unconsciousness. Additionally, animals were closely monitored post-surgery, and predefined humane endpoints were applied to prevent severe suffering.

All animals were housed in a specific pathogen free environment with a temperature of 23 °C ± 2 °C, relative humidity of 55% ± 2%, and 12 h light/dark cycle, with adequate food and water.

### 2.3 Animal grouping and treatment

In this study, 90 rats were randomly assigned to 6 groups, with 15 rats in each group. After 7 days of adaptive feeding, drugs were intraperitoneally injected once a day for 14 days. Ginsenoside Rg1 (Rg1) and salidroside (Sal) solutions were prepared with physiological saline, and freshly prepared for each administration. The specific groups were as follows: the sham group, MIRI group, Rg1 group (20 mg/kg), Sal group (40 mg/kg), PRC group (20 mg/kg Rg1 + 40 mg/kg Sal), and 3-MA group (15 mg/kg). The concentrations of ginsenoside Rg1 and salidroside were both based on previously published studies (Zhu et al., 2015; Chang et al., 2016; Chen et al., 2017; Xu et al., 2024). The sham group and MIRI group were administered an equal volume of physiological saline intraperitoneally every day.

### 2.4 Establishment of the rat MIRI model

After 14 days of drug administration, MIRI models were established in all rats, except those in the sham group. The MIRI model was prepared following the methods described in the literature (Katz et al., 2019; Qiu et al., 2024). Rats were fasted for 12 h before the procedure, with free access to water. General anesthesia was induced using an isoflurane gas anesthesia machine. The rats were placed in the supine position on a rat board, and electrocardiograms were continuously monitored. The chest and neck were shaved and disinfected with 75% alcohol and povidone-iodine, respectively. Tracheal intubation was performed, and the rats were connected to a small-animal ventilator. After respiratory stabilization, a skin incision was made at the third-fourth intercostal space on the left side, and the tissues were dissected layer by layer to expose the intercostal space. A thoracic spreader was inserted to fully expose the heart. The pericardium was opened, and the left anterior descending coronary artery was located. A 5–0 suture was used to ligate the artery 2–3 mm below the left auricle of the left ventricle. A PE-10 thin tube was placed between the entry and exit points of the needle. After successful ligation, the ischemic myocardial area became pale, and a significant elevation of the ST segment was observed on the electrocardiogram. After 30 min of ligation, the slipknot was loosened, the thin tube was removed, and reperfusion was initiated. At this point, the heart color returned to normal, and the ST segment on the electrocardiogram decreased compared with the pre-reperfusion levels. After 120 min of reperfusion, the modeling was completed. The sham group underwent the same procedures without ligation.

### 2.5 TTC staining

After sampling, the heart was thoroughly rinsed with physiological saline at 4 °C, blotted dry with filter paper, and then placed in a −80 °C freezer for 20 min. The heart was then cut into 5 slices along the long axis, from the apex to the base, using a scalpel. Each slice was approximately 2–3 mm thick to ensure a neat cut surface. The heart slices were placed in a centrifuge tube covered with light-proof tin foil, and TTC staining solution was added. The tube was incubated in a 37 °C water bath for 30 min, with the centrifuge tube gently shaken every 10 min to ensure full contact between the heart slices and the TTC solution. After staining, the slices were rinsed repeatedly with distilled water for 1 min and then fixed in 4% paraformaldehyde. After 24 h of fixation, the heart tissue was removed, blotted dry with filter paper, and the staining results were observed and photographed for analysis. The TTC staining solution reacts with dehydrogenase to produce a red color. In myocardial ischemia-reperfusion, when the tissue is ischemic and infarcted, the activity of dehydrogenase decreases, and the tissue appears grayish-white or white.

### 2.6 HE staining

After sampling, the fresh tissue was fixed in 4% paraformaldehyde for more than 24 h, trimmed, and placed in a dehydration box. The tissue was then dehydrated through a gradient alcohol series using a dehydrator, followed by treatment with alcohol-benzene, xylene, and other solvents, before being immersed in wax and embedded. The embedded tissue was trimmed into wax blocks and sectioned using a paraffin slicer to a thickness of 4 μm. The slices were flattened on a spreading machine, mounted on glass slides, and baked. HE staining was performed. The sections were stained with hematoxylin to highlight the nucleus and eosin to stain the cytoplasm and extracellular components. The sections were then dehydrated, sealed with neutral gum, and observed under an optical microscope (×200) to compare the pathological conditions of the myocardial tissues in each group. Photographs were taken for analysis. The staining results showed that the nuclei were blue, and the cytoplasm was red.

### 2.7 Transmission electron microscopy

Fresh myocardial tissue pieces measuring approximately 1 mm × 1 mm × 1 mm were cut and quickly fixed in an electron microscope fixative at 4 °C for 2 h. The tissues were rinsed three times with 0.1M phosphate buffer (PB, pH 7.4), followed by fixation with 1% osmium tetroxide at room temperature for 2 h, and rinsed again. The myocardial tissue was dehydrated through a graded series of ethanol solutions and then subjected to overnight infiltration with acetone and 812 embedding agents in different proportions. Subsequently, the tissue was treated with 812 embedding agents, placed in an embedding mold, and incubated overnight at 37 °C. Afterward, it was processed at 60 °C for 48 h to complete the embedding process. Ultrathin sections (60–80 nm) were cut using an ultrathin microtome, stained with uranium and lead salts, and dried overnight at room temperature. Finally, the ultrastructure of the myocardial tissue was observed, and images were collected and analyzed under a transmission electron microscope.

### 2.8 Enzyme-linked immunosorbent assay (ELISA)

Blood samples collected from the abdominal aorta of rats were left at room temperature for 30 min. The samples were centrifuged at 3,000 rpm for 15 min at 4 °C. The serum was carefully collected into cryotubes, aliquoted, and stored at −80 °C until further analysis. Following the manufacturer’s instructions, the levels of TXA2, PGI2, sTM, and sEPCR in the serum were measured using ELISA kits.

### 2.9 Cell culture

H9c2 cells (Rat, CVCL_0286, CL-0089) were purchased from Wuhan Procell Life Science & Technology Co., Ltd. (Wuhan, China). DMEM (cat. no. 11965092) and glucose-free DMEM (cat. no. 11966025) were obtained from Thermo Fisher Scientific (Massachusetts, USA). Fetal bovine serum (FBS, cat. no.FB25015) were purchased from Clark Bioscience (Virginia, USA). The H9c2 cells were cultured in a cell incubator at 37 °C with 5% CO_2_. The culture medium was refreshed every 2–3 days with complete medium. The complete medium consisted of DMEM supplemented with 10% FBS, and 1% penicillin-streptomycin. When the growth density of H9c2 cells reached 80%–90%, they were digested with 0.25% trypsin for subculture and used in subsequent experiments.

### 2.10 Establishment of a hypoxia/reoxygenation (H/R) cell model

Referring to relevant literature, the *in vitro* MIRI model was simulated using the H/R method (Liu et al., 2023; Yang J. et al., 2024; Zhao Y. et al., 2024). Briefly, when the confluence of H9c2 cells reached 90%, the medium was changed. The original medium was replaced with glucose-free, serum-free medium. The cells were incubated in a hypoxic environment at 37 °C with 1% O_2_, 5% CO_2_, and 94% N_2_ for 4 h. After the hypoxic phase, the original medium was removed and replaced with complete medium. The cells were then placed in a reoxygenated environment at 37 °C with 21% O_2_, 5% CO_2_, and 74% N_2_ for 2 h.a.

### 2.11 Cytotoxicity assay

H9c2 cells (3 × 10^3^ cells/well) were seeded in 96 - well plates and the culture plates were incubated in an incubator at 37 °C with 5% CO_2_ for 24 h. After discarding the original medium, the cells were treated with different doses of Rg1 (0, 10, 20, 40, 80, 100, 200, 300, 400, and 500 μM) and Sal (0, 10, 25, 50, 100, 200, and 300 μM) for 24 h. Then, the original medium was discarded, 10 μL of CCK-8 solution was added to each well, and the plates were incubated in the dark at 37 °C with 5% CO_2_ for 30 min. The absorbance at 450 nm was measured using a microplate reader. Cell viability was expressed as a percentage. The concentrations of ginsenoside Rg1 and salidroside were based on previously published studies (Liu et al., 2022; Yan et al., 2022; Tian et al., 2023; Yang B. et al., 2024) and supplemented with preliminary experimental verification.

### 2.12 Cell viability assay

H9c2 cells (3 × 10^3^ cells/well) were seeded in 96 - well plates and the culture plates were incubated in an incubator at 37 °C with 5% CO_2_ for 24 h. The H9c2 cells were divided into the control group, H/R group, Rg1 (80, 100, 200 µM) groups, and Sal (25, 50, 100 µM) groups. The cells in the control group were incubated under normal conditions. The H/R, Rg1 (80, 100, and 200 µM), and Sal (25, 50, and 100 µM) groups were subjected to the H/R protocol. The corresponding concentrations of Rg1 and Sal were added to the glucose-free and serum-free medium in each drug-treated group. After the reoxygenation phase, the original medium was discarded, and 10 μL of CCK-8 reagent was added to each well. The plates were incubated in the dark at 37 °C with 5% CO_2_ for 30 min. The absorbance at 450 nm was measured using a microplate reader. Cell viability was expressed as a percentage.

### 2.13 Measurement of LDH release

H9c2 cells were seeded into 6-well plates at a density of 2 × 10^5^ cells/well and incubated for 24 h at 37 °C under 5% CO_2_. The cells were then divided into the following experimental groups: Control, H/R, Rg1, Sal, PRC, and 3-MA. Cells in the Control group were maintained under normal culture conditions. All other groups were subjected to hypoxia/reoxygenation (H/R) injury. During the H/R insult, cells in the respective drug-treated groups were exposed to the indicated concentrations of Rg1, Sal, PRC (the combination of Rg1 and Sal), or 5 mmol/L 3-MA (Gao et al., 2016) in glucose- and serum-free medium. After the treatment, the culture supernatant was collected for LDH analysis. Following the manufacturer’s protocol, reagents and samples from each group were dispensed into designated blank, standard, sample, and control wells. The plate was then incubated at 37 °C for 15 min, followed by the addition of 20 µL of 2,4-dinitrophenylhydrazine to each well. After a further 15 min incubation at 37 °C, 200 µL of 0.4 mol/L NaOH solution was added to each well and mixed thoroughly. The reaction mixture was allowed to stand at room temperature for 5 min. Finally, the absorbance of each sample was measured at 440 nm using a microplate reader.

### 2.14 Measurement of intracellular ROS by flow cytometry

H9c2 cells were seeded in 6-well plates at a density of 2 × 10^5^ cells/well and incubated at 37 °C with 5% CO_2_ for 24 h. The cells were then divided into the following groups: Control, H/R, Rg1, Sal, PRC, and 3-MA. The modeling and drug treatments were performed as described in the preceding section (Section 2.13). After the reoxygenation phase, the medium was discarded, and the cells were detached using 0.25% trypsin, collected by centrifugation at 1,000 rpm for 5 min. The intracellular ROS probe DCFH-DA was diluted 1:1,000 in serum-free DMEM. A volume of 1 mL of the diluted probe solution was added to each cell sample, followed by incubation at 37 °C for 20 min. During the incubation, the samples were gently inverted and mixed at 5-min intervals. After incubation, the cells were centrifuged at 1,000 rpm for 5 min and washed three times with serum-free DMEM. Finally, intracellular ROS levels were quantified by flow cytometry, with 10,000 events collected per sample for analysis.

### 2.15 Measurement of SOD and MDA activity

H9c2 cells were seeded in 6-well plates at a density of 2 × 10^5^ cells/well and incubated at 37 °C with 5% CO_2_ for 24 h. The cells were then divided into the following groups: Control, H/R, Rg1, Sal, PRC, and 3-MA. After the respective treatments, cells were harvested for the assessment of oxidative stress markers. The activities of superoxide dismutase (SOD) and malondialdehyde (MDA) levels were measured using commercial assay kits. SOD activity was determined by the hydroxylamine method according to the manufacturer’s protocol. Briefly, the detection reagents were added sequentially to the samples, which were then incubated at 37 °C for 40 min. After adding the chromogenic agent and standing at room temperature for 10 min, absorbance was measured at 550 nm using a microplate reader. MDA content was assessed via the thiobarbituric acid (TBA) method. Sample aliquots were mixed with TBA reagent, heated at 95 °C for 40 min, cooled, and centrifuged at 4,000 rpm for 10 min. The absorbance of the supernatant was read at 532 nm.

### 2.16 Measurement of ATP content

H9c2 cells (2 × 10^5^ cells/well) were seeded into 6-well plates and incubated at 37 °C with 5% CO_2_ for 24 h. The cells were divided into the following groups: control, H/R, Rg1, Sal, PRC, and 3-MA. The modeling and drug treatment procedures were performed as described in the preceding section. After the reoxygenation phase, the cells were washed twice with PBS. Subsequently, 200 µL of lysis buffer was added to each well, and the cell lysates were collected. The lysates were centrifuged at 12,000 rpm and 4 °C for 5 min. The supernatant was collected for subsequent analysis. All reagents were thawed on ice, and the ATP standard solution was diluted with lysis buffer to generate a concentration gradient. The ATP working solution was prepared according to the manufacturer’s instructions. Then, 100 µL of the ATP detection working solution was added to each well of the assay plate and allowed to stand at room temperature for 5 min to eliminate background ATP. Finally, 20 µL of each sample supernatant was added to the respective wells, mixed rapidly, and the ATP content was measured using a chemiluminescence detector.

### 2.17 Measurement of the mitochondrial membrane potential (MMP)

The mitochondrial membrane potential assay kit was thawed at room temperature, and an appropriate amount of JC-1 working solution was prepared according to the manufacturer’s instructions and stored in the dark. After treatments, cells were washed twice with PBS. Then, 1 mL of JC-1 staining working solution along with 1 mL of complete medium was added to each well, and the plates were incubated at 37 °C for 20 min. Following incubation, the supernatant was aspirated, and the cells were washed twice with JC-1 staining buffer. Subsequently, 2 mL of complete medium was added, and the results were observed and recorded under a fluorescence microscope.

### 2.18 siRNA transfection and knockdown validation

To genetically validate the essential roles of SIRT1 and SIRT3 in PRC-mediated protection, siRNA-mediated knockdown was performed in H9c2 cells. H9c2 cells (2 × 10^5^ cells/well) were seeded in 6-well plates and transfected at 50%–60% confluence. Rat-specific siRNAs targeting SIRT1 (siSIRT1#1, siSIRT1#2 and siSIRT1#3) or SIRT3 (siSIRT3#1, siSIRT3#2 and siSIRT3#3), along with a non-targeting control siRNA (siControl, all from Sangon Biotech Co., Ltd., Shanghai, China), were transfected using Lipofectamine™ 3000 (cat. no. L3000150, Thermo Fisher Scientific, Massachusetts, USA) according to the manufacturer’s instructions. Briefly, siRNA (final concentration 50 nM) and Lipofectamine™ 3000 reagent were separately diluted in Opti-MEM (cat. no. 31085070, Thermo Fisher Scientific, Massachusetts, USA), combined, and incubated at room temperature for 20 min to form complexes before addition to the cells. After 6 h of transfection, the medium was replaced with complete DMEM. Cells were then cultured for an additional 42 h to achieve maximal protein knockdown before further experimental treatments. To confirm knockdown efficacy, parallel samples were harvested at 48 h post-transfection for Western blot analysis. For mechanistic validation, the following groups were established: Control (siControl, normal conditions), Model (siControl + H/R), PRC (siControl + H/R + 100 µM Rg1 + 25 µM Sal), siSIRT1+PRC (siSIRT1 + H/R + 100 µM Rg1 + 25 µM Sal), and siSIRT3+PRC (siSIRT3 + H/R + 100 µM Rg1 + 25 µM Sal). To determine whether the protective effects of PRC depended on SIRT1 or SIRT3, these groups were subjected to key functional assays after the respective H/R and drug treatments. Specifically, intracellular ROS levels and mitochondrial membrane potential (MMP) were assessed by flow cytometry (DCFH-DA, Section 2.14) and JC-1 staining (Section 2.17), respectively.

### 2.19 Western blotting

Protein expression was analyzed by Western blotting in: (1) cardiac tissue from MIRI rats (SIRT1, SIRT3, PGC-1α, NRF2, Beclin 1, p62, PINK1, Parkin, TOM20); (2) H9c2 cells (SIRT1, SIRT3, PGC-1α, NRF2, Beclin 1, p62); and (3) SIRT1/3-knockdown H9c2 cells (SIRT1, SIRT3, PGC-1α, NRF2, Beclin 1, p62, PINK1, Parkin, TOM20). Cardiac tissue proteins were extracted using RIPA lysis buffer containing protease and phosphatase inhibitors, and the protein concentration was determined using a BCA protein assay kit. Protein samples (30 µg) were mixed with 5× loading buffer at a ratio of 4:1 and boiled at 100 °C for 5 min. The proteins were separated by sodium dodecyl sulfate-polyacrylamide gel electrophoresis (SDS-PAGE) and transferred to a PVDF membrane. The membrane was blocked with a protein-free rapid blocking solution at room temperature for 10 min, followed by incubation overnight at 4 °C with the primary antibodies on a shaker. The primary antibodies used were: SIRT1 (Affinity, DF6033, Rabbit, Q96EB6, AB_2838007, 1:1000, Ohio, USA), SIRT3 (Proteintech, 10099-1-AP, Rabbit, BC001042, AB_2239240, 1:5000, Wuhan, China), PGC-1α (Proteintech, 66369-1-lg, Mouse, NM_013261, AB_2828002, 1:5000, Wuhan, China), NRF2 (Proteintech, 16396-1-AP, Rabbit, BC011558, AB_2782956, 1:5000, Wuhan, China), Beclin 1 (Proteintech, 11306-1-AP, Rabbit, BC010276, AB_2259061, 1:5000, Wuhan, China), p62 (Affinity, AF5384, Rabbit, Q13501, AB_2837869, 1:1000, Ohio, USA), PINK1 (Proteintech, 81991-4-RR, Rabbit, BC028215, AB_3670512, 1:5000, Wuhan, China), Parkin (Proteintech, 66674-1-Ig, Mouse, BC022014, AB_2882028, 1:5000, Wuhan, China) and TOM20 (Proteintech, 80501-1-RR, Rabbit, BC000882, AB_2918897, 1:5000, Wuhan, China). The PVDF membrane was then washed five times with TBST, and incubated with the corresponding secondary antibody (Proteintech, HRP Goat Anti-Mouse/Rabbit IgG (H + L) Antibody, SA00001-1, SA00001-2, AB_2722565, AB_2722564, 1:2000, Wuhan, China) at room temperature for 1 h. Afterward, the membrane was washed five times with TBST, and the bands were visualized using an ECL detection reagent. Finally, protein expression levels were analyzed using ImageJ software. The loading control was β-actin (Proteintech, 66009-1-lg, Mouse, NM_001101, AB_2697938, 1:50000, Wuhan, China).

### 2.20 Statistical analysis

All statistical analyses were conducted with GraphPad Prism 8.0.2. Data were manifested as mean ± standard deviation, sourced from a minimum of three independent replications. Unless otherwise specified, after confirming assumptions of normality (Shapiro-Wilk test) and equal variance (Levene’s test), one-way ANOVA with Tukey’s post hoc test was used for intergroup comparisons. If either assumption was not met, the Kruskal–Wallis test with Dunn’s post hoc test was utilized. A *P <* 0.05 was considered statistically significant.

## 3 Results

### 3.1 Effects of PRC on myocardial tissue morphology in MIRI rats

**FIGURE 1:**
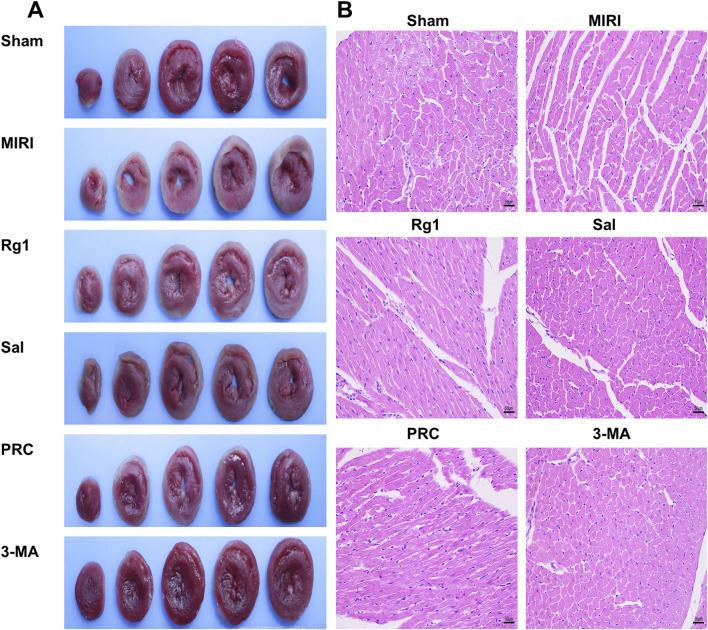
Effects of PRC on myocardial tissue morphology in MIRI rats. **(A)** Representative heart pictures of TTC staining. **(B)** Representative heart pictures of HE staining. Scale bar equals 50 µm.

As shown in Figure 1A, TTC staining results revealed no ischemic infarct area in the heart sections of the sham group, whereas a significant gray-white infarct zone was observed in the MIRI group. The gray-white infarct regions in the Rg1, Sal, PRC, and 3-MA groups were reduced to varying extents compared with those in the MIRI group. HE staining analysis, shown in Figure 1B, demonstrated that myocardial fiber bundles in the sham group were neatly organized, with minimal swelling of the muscle cells. There was no significant infiltration of inflammatory cells, and the overall myocardial architecture appeared normal. In contrast, the MIRI group exhibited substantial tissue expansion in the myocardial infarct area, accompanied by edema. The myofibril sarcomeres were disrupted, myocardial cells were hypertrophied, and numerous inflammatory cells were recruited and infiltrated into the myocardial tissue. In addition, local necrotic foci and fibroblast proliferation were observed. In the Rg1 and Sal groups, myocardial cells were hypertrophied but displayed a more orderly arrangement than those in the MIRI group, with fewer ruptured myocardial fibers and significantly less inflammatory cell infiltration.The PRC group and 3-MA group further improved myocardial myofibril sarcomeres, reduced inflammatory cell infiltration, and mitigated MIRI-induced damage to cardiomyocytes.

### 3.2 Effects of PRC on the ultrastructure of myocardial mitochondria in MIRI rats

**FIGURE 2:**
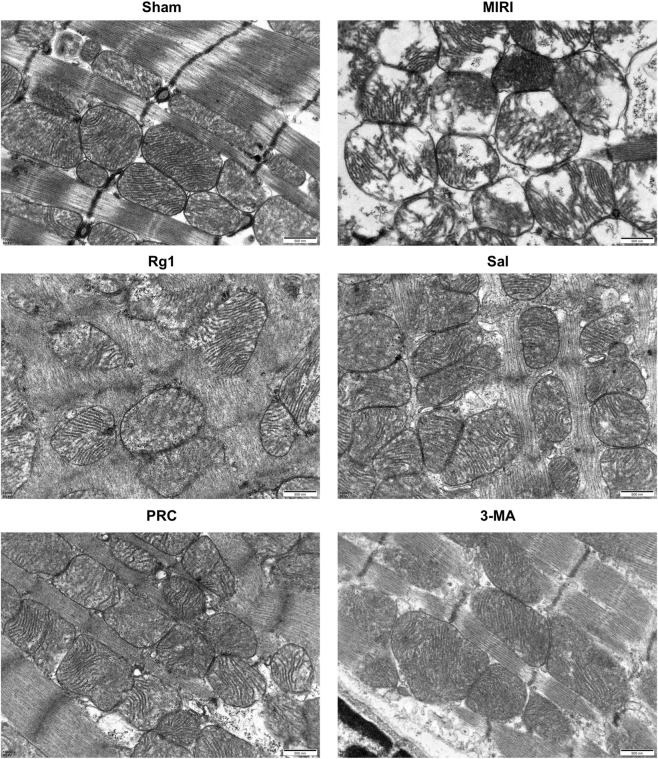
Effects of PRC on the ultrastructure of myocardial mitochondria in MIRI rats. Scale bar equals 500 nm.

Transmission electron microscopy of the mitochondrial ultrastructure revealed that myocardial tissue in the sham group was organized into bundles with uniform density, and the mitochondrial double-membrane structure was well-defined. In the MIRI group, mitochondria were swollen and deformed, with ruptured membranes, increased membrane density, and extensive vacuolization. In the Rg1, Sal, PRC, and 3-MA groups, the myocardial tissue remained relatively organized, with fewer damaged mitochondrial structures and significantly reduced mitochondrial swelling and vacuolization. The specific results are presented in Figure 2.

### 3.3 Effects of PRC on the endothelial function of myocardial blood vessels in MIRI rats

**FIGURE 3:**
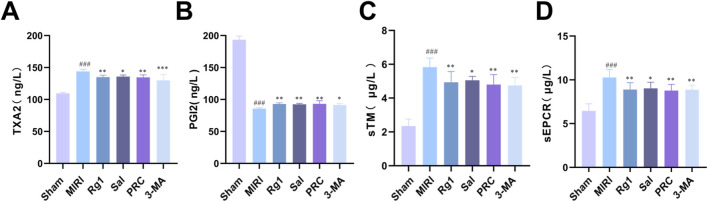
Effects of PRC on the endothelial function of myocardial blood vessels in MIRI rats. **(A–D)** Biomolecules related to endothelial function, including TXA2, PGI2, sTM, and sEPCR, were quantified by ELISA (n = 8). Data values are depicted as mean ± standard deviation. ^###^ *P* < 0.001 vs. Sham group; ^*^ *P* < 0.05, ^**^ *P* < 0.01, ^***^ *P* < 0.001 vs. MIRI group.

As shown in Figure 3, the MIRI group exhibited significantly increased TXA2 content compared with the sham group (*P* < 0.001). TXA2 levels were significantly reduced in the Rg1, Sal, PRC, and 3-MA groups compared with those in the MIRI group. Additionally, PGI2 content was markedly lower in the MIRI group compared with the sham group (*P* < 0.001), whereas PGI2 levels were significantly higher in the Rg1, Sal, PRC, and 3-MA groups relative to the MIRI group. Similarly, sTM and sEPCR levels were significantly elevated in the MIRI group compared with the sham group *(P* < 0.001), and significantly reduced in the Rg1, Sal, PRC, and 3-MA groups compared with the MIRI group.

### 3.4 Effects of PRC on the cytotoxicity and viability of H9c2 cells

**FIGURE 4:**
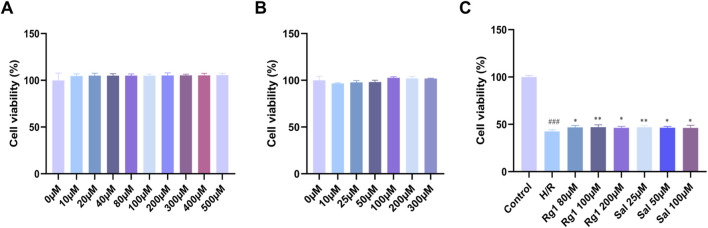
Effects of PRC on the cytotoxicity and viability of H9c2 cells. **(A)** The effect of different concentrations of ginsenoside Rg1 on H9c2 cell viability was determined by CCK-8 assay (n = 7). **(B)** The effect of different concentrations of salidroside on H9c2 cell viability was determined by CCK-8 assay (n = 6). **(C)** The effects of different concentrations of ginsenoside Rg1 or salidroside on the viability of H/R-treated H9c2 cell was determined by the CCK-8 assay (n = 6). Data values are depicted as mean ± standard deviation. ^###^ *P* < 0.001 vs. Control group; ^*^ *P* < 0.05, ^**^ *P* < 0.01 vs. H/R group.

As shown in Figures 4A,B, after treating H9c2 cells with various concentrations of Rg1 (0, 10, 20, 40, 80, 100, 200, 300, 400, 500 µM) and Sal (0, 10, 25, 50, 100, 200, 300 µM) for 24 h, no significant changes in cell survival rates were observed across the groups (*P* > 0.05), indicating that ginsenoside Rg1 (0–500 µM) and salidroside (0–300 µM) did not exhibit cytotoxic effects on H9c2 cells under normal culture conditions. Figure 4C shows that, compared with the control group, cell activity in the H/R group was significantly reduced (*P* < 0.001). Compared with the H/R group, ginsenoside Rg1 at concentrations of 80, 100, and 200 µM significantly increased cell activity (*P* < 0.05, *P* < 0.01, *P* < 0.05), with the highest survival rate observed at 100 µM. Similarly, salidroside at concentrations of 25, 50, and 100 µM significantly increased cell activity compared with the H/R group (*P* < 0.01, *P* < 0.05, *P* < 0.05), with the highest survival rate at 25 µM. Based on these results, 100 µM ginsenoside Rg1 and 25 µM salidroside were selected for subsequent experiments.

### 3.5 Effects of PRC on oxidative stress

**FIGURE 5:**
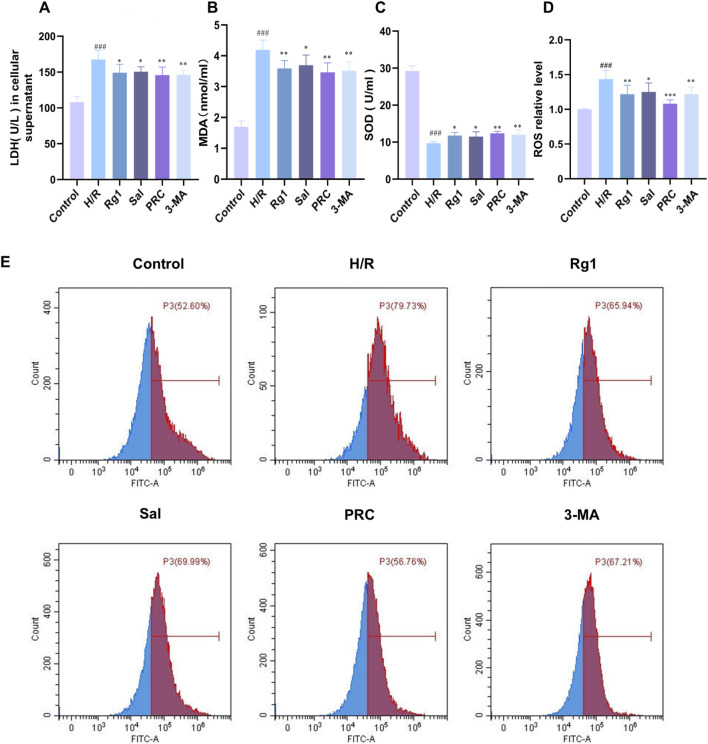
Effects of PRC on oxidative stress. **(A)** Detection of LDH levels in cell supernatants (n = 6). **(B)** Detection of MDA levels by a kit (n = 6). **(C)** Detection of SOD activity by a kit (n = 6). **(D,E)** ROS levels were detected by flow cytometry (n = 7). Data values are depicted as mean ± standard deviation. ^###^ *P* < 0.001 vs. Control group; ^*^ *P* < 0.05, ^**^ *P* < 0.01, ^***^ *P* < 0.001 vs. H/R group.

Compared with the control group, the H/R group exhibited significantly increased LDH leakage, MDA, and ROS levels in H9c2 cells (*P* < 0.001), while SOD content was significantly decreased (*P* < 0.001). In contrast, the Rg1, Sal, PRC, and 3-MA groups showed significantly decreased levels of LDH leakage, MDA, and ROS, along with increased SOD content compared with the H/R group. The specific results are shown in Figure 5.

### 3.6 Effects of PRC on the mitochondrial function of cardiomyocytes

**FIGURE 6:**
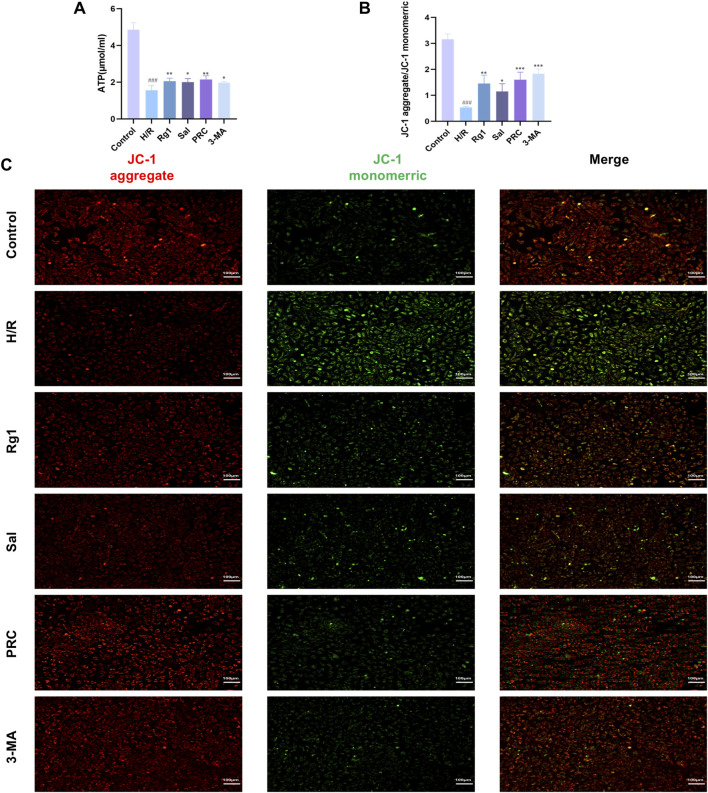
Effects of PRC on the mitochondrial function of cardiomyocytes. **(A)** Detection of ATP levels by a kit (n = 6). **(B,C)** Mitochondrial membrane potential was detected using the fluorescent dye JC-1 (n = 3). Scale bar equals 100 µm. Data values are depicted as mean ± standard deviation. ^###^ *P* < 0.001 vs. Control group; ^*^ *P* < 0.05, ^**^ *P* < 0.01, ^***^ *P* < 0.001 vs. H/R group.

Compared with the control group, ATP content and mitochondrial membrane potential in H9c2 cells were significantly reduced in the H/R group (*P* < 0.001). In the Rg1, Sal, PRC, and 3-MA groups, ATP content and mitochondrial membrane potential were significantly higher compared with the H/R group. The specific results are shown in Figure 6.

### 3.7 Effects of PRC on the expression levels of SIRT1, SIRT3, PGC-1α, and NRF2 proteins in myocardial tissue of MIRI rats

**FIGURE 7:**
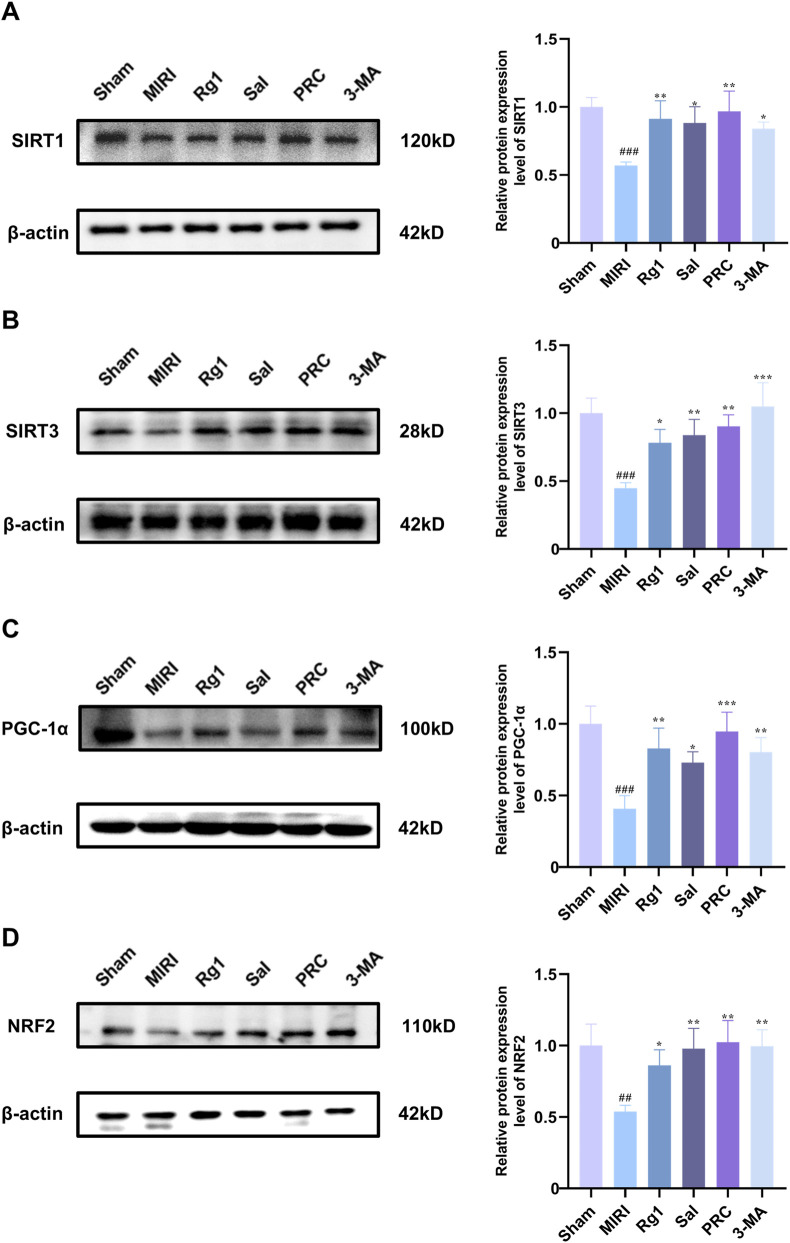
Effects of PRC on the expression levels of SIRT1, SIRT3, PGC-1α, and NRF2 proteins in myocardial tissue of MIRI rats. **(A–D)** WB detection and quantification analysis of SIRT1, SIRT3, PGC-1α, and NRF2 (n = 3). Data values are depicted as mean ± standard deviation. ^###^ *P* < 0.001 vs. Sham group; ^*^ *P* < 0.05, ^**^ *P* < 0.01, ^***^ *P* < 0.001 vs. MIRI group.

To elucidate the underlying molecular mechanism, we examined the expression of key proteins in the SIRT1/3-PGC-1α-NRF2 pathway in myocardial tissue. As demonstrated in Figure 7, the expression levels of SIRT1, SIRT3, PGC-1α, and NRF2 in myocardial tissue were significantly lower in the MIRI group compared with the sham group (*P* < 0.001). However, the expression levels of these proteins were significantly higher in the Rg1, Sal, PRC, and 3-MA groups than in the MIRI group.

### 3.8 Effects of PRC on the expression levels of SIRT1, SIRT3, PGC-1α, and NRF2 in H9c2 cells

**FIGURE 8:**
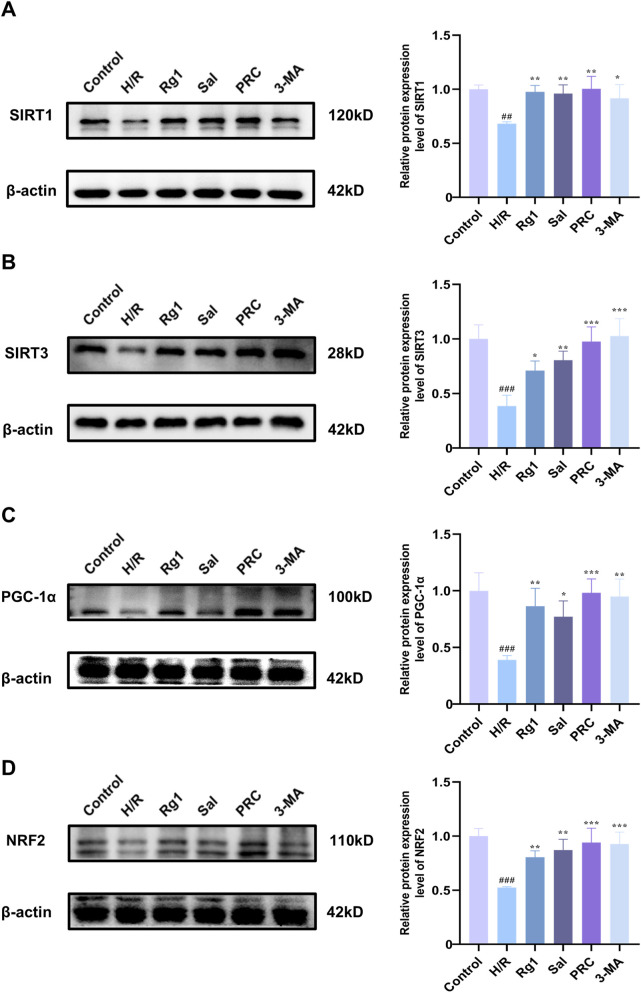
Effects of PRC on the expression levels of SIRT1, SIRT3, PGC-1α, and NRF2 in H9c2 cells. **(A–D)** WB detection and quantification analysis of SIRT1, SIRT3, PGC-1α, and NRF2 (n = 3). Data values are depicted as mean ± standard deviation. ^###^ *P* < 0.001 vs. Control group; ^*^ *P* < 0.05, ^**^ *P* < 0.01, ^***^ *P* < 0.001 vs. H/R group.

As demonstrated in Figure 8, compared with the control group, the expression levels of SIRT1, SIRT3, PGC-1α, and NRF2 in H9c2 cells of the H/R group were significantly lower (*P* < 0.001). However, in the Rg1, Sal, PRC, and 3-MA groups, the expression levels of these proteins were significantly higher compared with the H/R group.

### 3.9 Effects of PRC on the expression levels of autophagy-related proteins in myocardial tissue of MIRI rats

**FIGURE 9:**
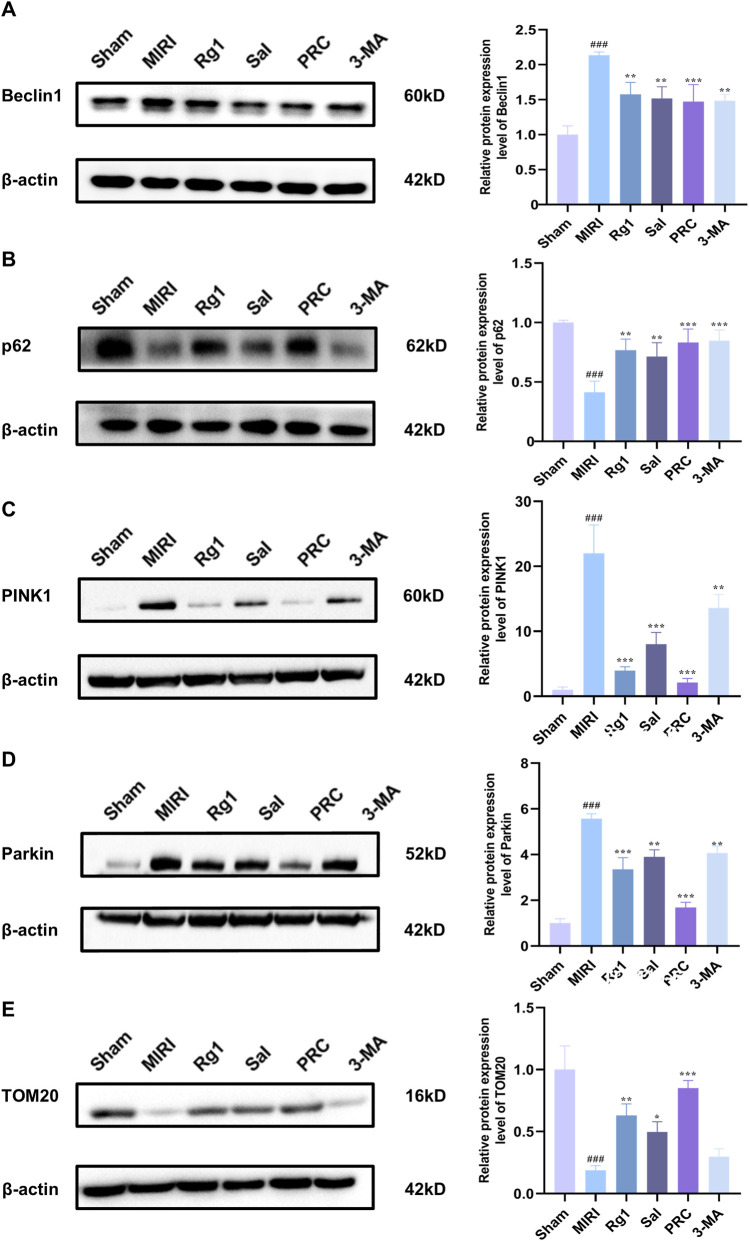
Effects of PRC on the expression levels of autophagy-related proteins in myocardial tissue of MIRI rats. **(A–E)** WB detection and quantification analysis of Beclin 1, p62, PINK1, Parkin, and TOM20 (n = 3). Data values are depicted as mean ± standard deviation. ^###^ *P* < 0.001 vs. Control group; ^*^ *P* < 0.05, ^**^ *P* < 0.01, ^***^ *P* < 0.001 vs. H/R group.

As shown in Figure 9, Western blot analysis of myocardial tissue revealed significant dysregulation of autophagy and mitophagy pathways following MIRI. The expression level of the autophagy marker Beclin 1 was significantly higher in the MIRI group than in the sham group (*P <* 0.001), while the autophagic substrate p62 was significantly lower (*P <* 0.001). Consistent with the induction of excessive mitophagy, the protein levels of the key mitophagy initiators PINK1 and Parkin were also markedly elevated in the MIRI group compared to the sham group (*P <* 0.001). Treatment with Rg1, Sal, PRC, or the autophagy inhibitor 3-MA effectively reversed these alterations. Compared with the MIRI group, these treatment groups showed a significant decrease in Beclin 1 expression, a significant increase in p62 accumulation, and a pronounced downregulation of both PINK1 and Parkin levels. Conversely, the expression trend of the mitochondrial protein TOM20 was opposite to that of PINK1 and Parkin: it was significantly reduced by MIRI (*P <* 0.001) and restored by the treatments.

### 3.10 Effects of PRC on the expression levels of autophagy-related proteins Beclin 1 and p62 in H9c2 cells

**FIGURE 10:**
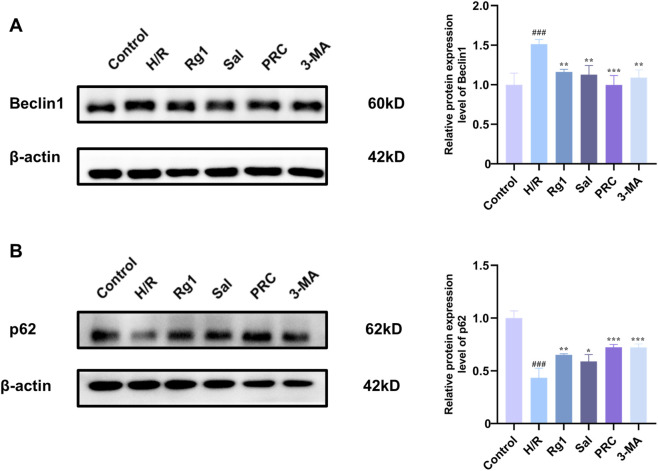
Effects of PRC on the expression levels of autophagy-related proteins Beclin 1 and p62 in H9c2 cells. **(A,B)** WB detection and quantification analysis of Beclin 1 and p62 (n = 3). Data values are depicted as mean ± standard deviation. ^###^ *P* < 0.001 vs. Sham group; ^*^ *P* < 0.05, ^**^ *P* < 0.01, ^***^ *P* < 0.001 vs. MIRI group.

As shown in Figure 10, compared with the control group, the expression level of Beclin 1 in H9c2 cells of the H/R group was significantly increased (*P* < 0.001), while the expression level of p62 was significantly decreased (*P* < 0.001). In contrast, in the Rg1, Sal, PRC, and 3-MA groups, the expression level of Beclin 1 was significantly lower, and the expression level of p62 was significantly higher compared with the H/R group.

### 3.11 siSIRT1 or siSIRT3 transfection abolishes the protective effects of PRC

**FIGURE 11:**
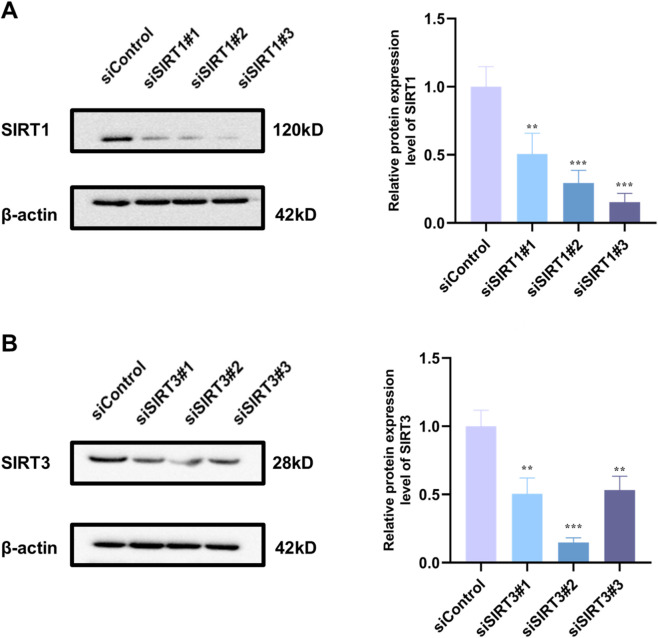
Validation of SIRT1 and SIRT3 knockdown efficiency by siRNA in H9c2 cells. **(A)** Representative blots and quantification of SIRT1 protein expression (n = 3). **(B)** Representative blots and quantification of SIRT3 protein expression (n = 3). Data values are depicted as mean ± standard deviation. ^*^ *P* < 0.05, ^**^ *P* < 0.01, ^***^ *P* < 0.001.

**FIGURE 12:**
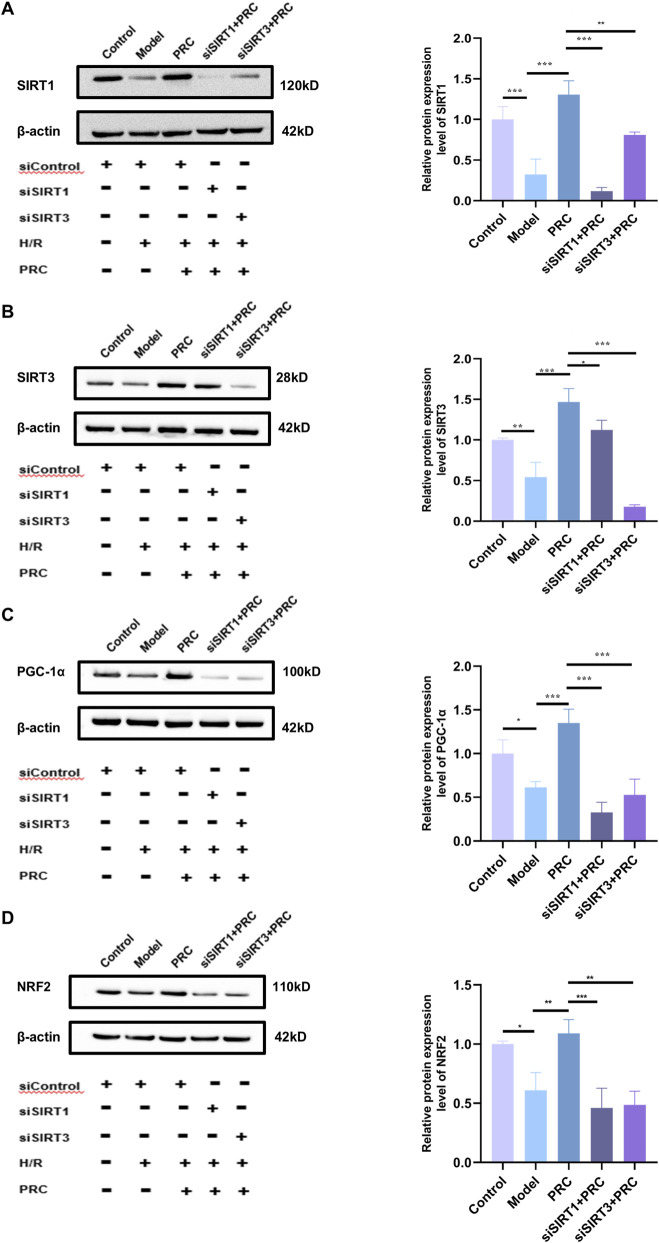
Effect of SIRT1 or SIRT3 knockdown on the abrogation of PRC-induced activation of the SIRT1/3-PGC-1α-NRF2 pathway. **(A–D)** WB detection and quantification analysis of SIRT1, SIRT3, PGC-1α, and NRF2 (n = 3). Data values are depicted as mean ± standard deviation. ^*^ *P* < 0.05, ^**^ *P* < 0.01, ^***^ *P* < 0.001.

**FIGURE 13:**
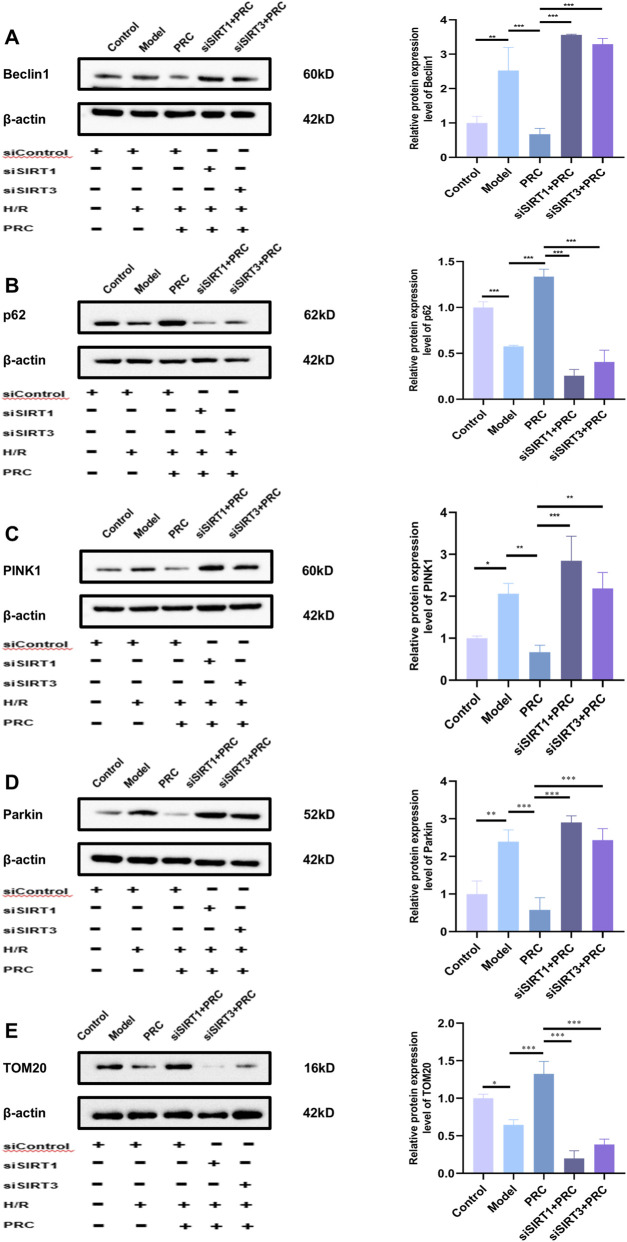
Effect of SIRT1 or SIRT3 knockdown on the abrogation of PRC-induced regulation of autophagy-related proteins. **(A–E)** WB detection and quantification analysis of Beclin 1, p62, PINK1, Parkin, and TOM20 (n = 3). Data values are depicted as mean ± standard deviation. ^*^ *P* < 0.05, ^**^ *P* < 0.01, ^***^ *P* < 0.001.

**FIGURE 14:**
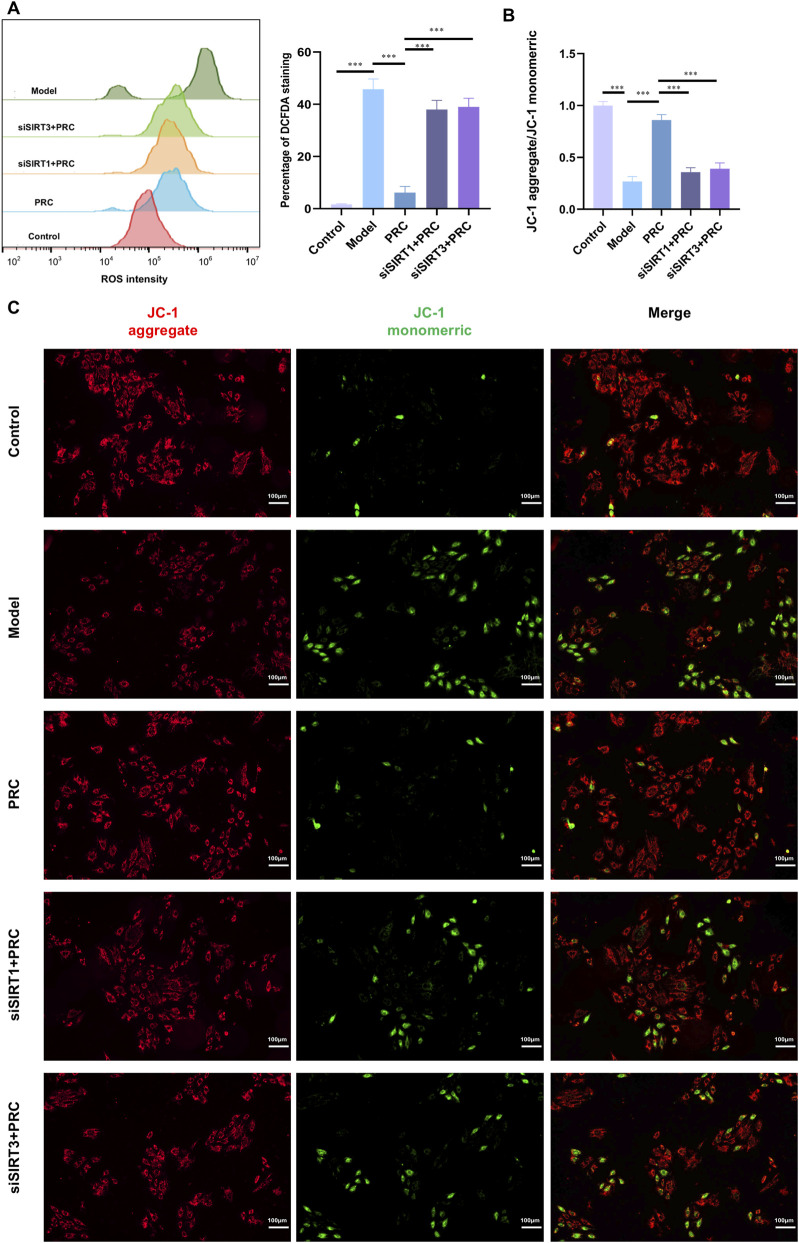
siSIRT1 or siSIRT3 transfection abolishes the protective effects of PRC. **(A)** Intracellular ROS levels were detected by flow cytometry using DCFH-DA staining (n = 3). **(B,C)** Mitochondrial membrane potential was assessed using the fluorescent dye JC-1. (n = 3). Scale bar equals 100 µm. Data values are depicted as mean ± standard deviation. ^*^ *P* < 0.05, ^**^ *P* < 0.01, ^***^ *P* < 0.001.

As shown in Figure 11, siSIRT1#3 potently suppressed SIRT1 protein expression, whereas siSIRT3#2 effectively abrogated SIRT3 protein production, verifying the successful establishment of target gene knockdown models. In siControl-transfected cells, PRC treatment significantly upregulated the expression of key signaling components, including SIRT1, SIRT3, PGC-1α, and NRF2, relative to the Model group (Figure 12). Concomitantly, PRC modulated the expression of autophagy- and mitophagy-related markers by downregulating Beclin 1, PINK1, and Parkin while upregulating p62 and preserving mitochondrial content as reflected by increased TOM20 levels (Figure 13). Importantly, all of these PRC-induced molecular alterations were completely abolished when either SIRT1 or SIRT3 was knocked down, as shown in the siSIRT1+PRC and siSIRT3+PRC groups. Furthermore, SIRT1/3 knockdown abolished the functional protective effects of PRC. Specifically, the PRC-induced attenuation of ROS production and restoration of mitochondrial membrane potential (MMP) in siControl-transfected cells were markedly abrogated in both SIRT1-and SIRT3-knockdown groups (Figure 14). These data provide unambiguous genetic evidence that SIRT1 and SIRT3 are essential for both the molecular signaling and functional protection conferred by PRC.

## 4 Discussion

Mitochondrial dysfunction is a central pathological event in MIRI (Sun et al., 2021; Zhang et al., 2023). This study demonstrates that PRC significantly alleviates MIRI. The core mechanism involves PRC’s regulation of the SIRT1/3-PGC-1α-NRF2 signaling axis to inhibit excessive mitophagy, thereby restoring mitochondrial quality control. This upstream, homeostatic mode of action is conceptually distinct from the direct autophagic inhibition by agents like 3-MA.

This study provides consistent evidence of the protective effects of PRC in both *in vivo* and *in vitro* models. PRC administration significantly reduced myocardial infarct size, improved myocardial fiber arrangement, attenuated inflammatory infiltration, and improved vascular endothelial function in MIRI rats. At the subcellular level, PRC mitigated mitochondrial swelling and vacuolization. These morphological improvements were associated with functional restoration, including increased ATP production, enhanced mitochondrial membrane potential (MMP), and reduced oxidative stress in H9c2 cells subjected to H/R injury. These findings establish the multi-level protective efficacy of PRC, demonstrating its coordinated protection on both cardiomyocytes and vascular endothelium, laying the groundwork for subsequent investigations into its underlying mechanism.

Autophagic dysregulation was identified as a critical bridge linking MIRI to mitochondrial damage (Wang and Ren, 2018; Chen et al., 2021). PRC effectively suppressed excessive autophagy by downregulating Beclin 1 and upregulating p62, an effect consistently demonstrated in both myocardial tissues and H9c2 cells. This correction of autophagic flux was directly correlated with the amelioration of mitochondrial ultrastructural damage and the recovery of bioenergetic function. Crucially, our *in vivo* data further revealed that PRC concomitantly suppressed the aberrant upregulation of the key mitophagy initiators PINK1 and Parkin while preserving mitochondrial content (indicated by TOM20), indicating a parallel fine-tuning of both bulk autophagy and selective mitophagy. Notably, while the autophagy inhibitor 3-MA also conferred protection, we propose that PRC operates through a fundamentally distinct, upstream mechanism. Unlike 3-MA, which directly and non-selectively inhibits the autophagic machinery by targeting Vps34 and preventing phagophore formation (Stencel et al., 2025), PRC appears to function as a ‘homeostatic regulator’ that fine-tunes mitophagy by modulating cellular signaling pathways. This body of evidence establishes a clear causal chain: PRC inhibits excessive autophagy, which in turn improves mitochondrial morphology and function, ultimately contributing to cardiomyocyte protection. This finely-tuned regulation of autophagy is crucial, as it prevents the detrimental effects of both insufficient and excessive mitophagy.

Our findings delineate a hierarchical regulatory architecture within the SIRT1/3-PGC-1α-NRF2 pathway during MIRI, wherein SIRT1 and SIRT3 serve as essential upstream modulators. Genetic evidence from siRNA knockdown experiments is conclusive: ablation of either SIRT1 or SIRT3 abolished PRC-elicited activation of the downstream PGC-1α-NRF2 signaling axis, normalization of PINK1/Parkin expression profiles, and restoration of mitochondrial structural and functional integrity. Most importantly, these molecular perturbations translated to tangible functional deficits; the PRC-mediated attenuation of ROS production and recovery of mitochondrial membrane potential were similarly abrogated following SIRT1/3 silencing. This genetic validation establishes SIRT1/3 as the critical upstream regulatory node through which PRC fine-tunes mitophagy dynamics, in contrast to the direct downstream inhibitory effects of agents such as 3-MA. The activated PGC-1α-NRF2 axis downstream of SIRT1/3 promotes mitochondrial biogenesis and antioxidant defense, which aligns with previous reports on SIRT1-mediated modulation of PGC-1α activity and SIRT3-dependent maintenance of mitochondrial homeostasis (Nemoto et al., 2005; Kong et al., 2010). Beyond this, the axis also contributes to suppressing excessive autophagy under stress (Wang et al., 2022; Liu et al., 2024). A direct molecular link to the autophagy machinery is further supported by the known ability of SIRT1 to deacetylate core autophagy-related proteins such as Atg5, Atg7, LC3 (Lee et al., 2008). A key novel insight from our study is the functional connection established between this pathway and the PINK1/Parkin-mediated mitophagy system. We demonstrate that the ability of PRC to normalize aberrant PINK1/Parkin expression and preserve mitochondrial content (indicated by TOM20) is strictly dependent on SIRT1/3. This *in vivo* evidence, showing parallel fine-tuning of both bulk autophagy markers and selective mitophagy initiators, reinforces that PRC acts through SIRT1/3 to calibrate an integrated mitochondrial quality control program. Collectively, our results demonstrate that the SIRT1/3 axis serves as an essential upstream integrator, coordinating PGC-1α-NRF2-mediated mitochondrial renewal with PINK1/Parkin-mediated precision clearance. The elucidation of this coordinated, multi-targeted regulatory mechanism provides a novel theoretical framework for understanding how combined natural compounds confer protection in complex diseases like MIRI (Zhang et al., 2023; Guo et al., 2024).

This study has limitations that point to directions for future research. First, regarding mechanistic depth, while our genetic knockdown data definitively establish SIRT1/3 as essential upstream regulators, the use of a PRC + 3-MA co-treatment could have further delineated the signaling hierarchy relative to the point of autophagic inhibition. Second, on the path to translational relevance, our findings would be strengthened by future studies that establish detailed dose-response relationships for PRC and, crucially, incorporate pharmacokinetic analyses to determine the achieved plasma and tissue concentrations of its active components. These steps are vital for bridging the gap between our preclinical models and potential clinical application.

In conclusion, our study elucidates that PRC ameliorates MIRI primarily by activating the SIRT1/3–PGC-1α–NRF2 axis and subsequently calibrating PINK1/Parkin-mediated mitophagy. This homeostatic mechanism, which restores upstream metabolic and redox balance, is fundamentally distinct from direct autophagy inhibition (e.g., by 3-MA). The concurrent benefits on vascular endothelial function further underscore its multi-targeted nature. Together, this integrated strategy, which simultaneously promotes mitochondrial biogenesis, enhances antioxidant capacity, and fine-tunes mitochondrial quality control, highlights the unique therapeutic advantage of combined natural product formulations against MIRI.

## Data Availability

The raw data supporting the conclusions of this article will be made available by the authors, upon reasonable request.
